# Disentangling Associations Among Maternal Lifetime and Prenatal Stress, Psychological Functioning During Pregnancy, Maternal Race/Ethnicity, and Infant Negative Affectivity at Age 6 Months: A Mixtures Approach

**DOI:** 10.1089/heq.2020.0032

**Published:** 2020-11-16

**Authors:** Rebecca K. Campbell, Paul Curtin, Michelle Bosquet Enlow, Kelly J. Brunst, Robert O. Wright, Rosalind J. Wright

**Affiliations:** ^1^ Department of Pediatrics, Kravis Children's Hospital, Icahn School of Medicine at Mount Sinai, New York, New York, USA.; ^2^ Department of Environmental Medicine and Public Health, Icahn School of Medicine at Mount Sinai, New York, New York, USA.; ^3^ Department of Psychiatry, Boston Children's Hospital, Boston, Massachusetts, USA.; ^4^ Department of Psychiatry, Harvard Medical School, Boston, Massachusetts, USA.; ^5^ Department of Environmental and Public Health Sciences, University of Cincinnati College of Medicine, Cincinnati, Ohio, USA.; ^6^ Institute for Exposomic Research, Icahn School of Medicine at Mount Sinai, New York, New York, USA.

**Keywords:** maternal stress, pregnancy, infant temperament, depression, post-traumatic stress disorder, race/ethnicity, mixtures, weighted quantile sum regression

## Abstract

**Purpose:** Maternal stress and psychological dysfunction in pregnancy are independently linked with fetal neurodevelopment. Stress encompasses environmental stressors and psychological and physiological responses. Stressors and psychopathology co-occur with patterns differing by race/ethnicity. We aimed to extend environmental mixtures methodology to elucidate prenatal stress associations with infant negative affectivity (NA) in a racially/ethnically mixed cohort.

**Methods:** Participants were mother/infant dyads (*n*=445) in a prospective pregnancy cohort study in two urban US settings in 2011–2018. During pregnancy, women completed the Life Stressor Checklist-Revised, Crisis in Family Systems-Revised, Edinburgh Postnatal Depression Scale, and post-traumatic stress disorder (PTSD) Checklist-Civilian version; the Infant Behavior Questionnaire-Revised assessed NA in 6-month olds. Using weighted quantile sum (WQS) regression, we developed a weighted maternal stress index encompassing lifetime and current life events and symptoms of depression and PTSD. Stress-by-race/ethnicity interactions allowed differential contributions of individual stress domains by maternal race/ethnicity.

**Results:** Mothers were majority black (44%) or Hispanic (37%). Stress questionnaire and infant NA scores were similar by race/ethnicity. The WQS prenatal stress score was positively associated with infant NA (*β*: 0.40 [95% confidence interval 0.16–0.64]). PTSD was the strongest contributor to the WQS score in Hispanic women (59%), whereas in black women, lifetime stress and depressive symptoms accounted for 38% and 35%, respectively, of the association with NA.

**Conclusions:** Extending environmental mixtures methodology to stress research may disentangle complex associations among lifetime and current stressful life events and psychological symptomatology and their contributions to early childhood neurobehavioral outcomes. Consideration of effect modification by race/ethnicity may inform understanding of differing vulnerability across racial/ethnic groups.

## Introduction

Maternal prenatal stress is a complex phenomenon encompassing the environmental stressors that women experience as well as their consequent psychological and physiological responses, which in turn can influence fetal neurodevelopment.^1,2^ In addition to stress experienced during pregnancy, chronic adversity and traumatic stressors experienced over the mother's lifetime are important to understanding effects on child health.^3–5^ Studies that include multiple measures of stress (traumatic and nontraumatic negative life events [NLEs], current vs. remote events, distress symptoms) considered one at a time show differential impacts of these various aspects of maternal prenatal stress on child behavioral outcomes.^1,6,7^

While the prevalence of neurobehavioral disorders has not been widely studied in socioeconomically and racially/ethnically diverse samples, there are data showing that black and Hispanic populations in the United States may be disproportionately burdened.^6,8^ Differential exposure to toxic environmental factors may influence these observed disparities. In the United States, cumulative exposure to both traumatic and nontraumatic stressors over a woman's lifetime differs by race and ethnicity, with minorities experiencing more adverse events compared with white women.^6,9^ Racial and ethnic minorities also experience unique sources of stress, such as discrimination, chronic financial strain, and higher prevalence of violence in their communities, which may enhance cumulative effects.^10–17^ Also, it has been shown that while the psychological sequelae of chronic stress are hypothesized to be biologically based (e.g., related to genetics or disruption of stress response systems such as the hypothalamic-pituitary-adrenal axis), they are also impacted by experience, including cultural and sociodemographic influences.^18^ Research demonstrates that chronic stress may manifest in different psychological symptom profiles across racial/ethnic groups, with documented variations in depressive, anxiety and post-traumatic stress disorder (PTSD) symptoms.^19,20^ Thus, when examining the types of stressful events as well as the associated psychological sequelae in diverse samples, methods that allow concurrent evaluation of measures across various stress components are needed.

With the expanding diversity of the US population and well-documented stress and health disparities for racial/ethnic minorities, methods that utilize a more integrated approach to disentangling these complexities may help identify those at increased risk for prenatal stress effects on adverse neurobehavioral outcomes in offspring. Specifically, extending methods used to study the health effects of environmental chemical mixtures^21,22^ to stress research may be an informative approach. Mixture approaches that evaluate the individual and joint relationships between multiple correlated exposures and outcomes have been increasingly used in environmental epidemiology to examine the health effects of classes of chemicals that cluster or co-occur (e.g., phthalates, metals). A similar conceptual approach can be applied to studies assessing correlated measures of the prenatal stress experience that may manifest in varying profiles across racial/ethnic groups.

In the current analyses, we applied weighted quantile sum (WQS) regression, a supervised method that models the total association between an exposure mixture and an outcome and quantifies the contribution of each mixture element to the overall relationship,^23^ to assess the additive effects and relative contribution of maternal stress and psychological dysfunction exposures to the outcome of interest, infant temperament. We aimed to develop a weighted prenatal stress index encompassing lifetime and current traumatic and nontraumatic life events, and domains of psychological functioning in pregnant women (depressive and PTSD symptoms). Studies show infant temperament is a stable characteristic (i.e., it predicts adult temperament) that also predicts social and neurocognitive functioning and behavioral and emotional problems later in life.^24^ We focused specifically on infant negative affectivity (NA), an indicator of greater proclivity toward expressions of fear, sadness, and distress, given that it is a widely studied temperament domain with prior research showing that it is a durable characteristic of temperament,^24,25^ that it predicts significant psychological dysfunction later in life^26^ and it predicts susceptibility to other adverse exposures.^27,28^ Furthermore, studies by our group and others linking prenatal maternal stress and psychological dysfunction to infant temperament have focused on the NA domain.^1,29,30^ We hypothesized that WQS regression would identify a mixture of stressors that predicts greater infant NA, and that the relative contributions of maternal stress and psychological dysfunction domains to infant NA would vary by maternal race/ethnicity.

## Methods

### Cohort selection

Participants were from the Programming of Intergenerational Stress Mechanisms (PRISM) study, an urban, longitudinal, pregnancy cohort study based in the northeastern United States, designed to investigate associations of prenatal psychosocial stress and other environmental exposures with child health outcomes, including neurobehavioral development. Pregnant English- and Spanish-speaking women aged 18 years and older carrying a singleton fetus were recruited at 22.5±9.0 weeks of gestation from prenatal clinics at the Beth Israel Deaconess Medical Center and the East Boston Neighborhood Health Center in Boston, Massachusetts (2011–2013) and the Mount Sinai Hospital in New York City (2013–2018). Study procedures were approved by the relevant Institutional Review Boards (Brigham and Women's Hospital/Partners HealthCare, Beth Israel Deaconess Medical Center, Icahn School of Medicine at Mount Sinai). Participants provided written informed consent in their preferred language. Study procedures were approved by the Institutional Review Boards of Brigham and Women's Hospital/Partners Health Care, Beth Israel Deaconess Medical Center and the Icahn School of Medicine at Mount Sinai.

### Exposures

Maternal stress and psychological functioning questionnaires were administered during pregnancy in a face-to-face interview in a private setting by trained research staff within ∼2 weeks of enrollment.

#### Lifetime stress

Lifetime stressful and potentially traumatic life events (including child maltreatment, interpersonal violence, sexual assault) were assessed with the Life Stressor Checklist—Revised (LSC-R).^31^ For each endorsed item in the 30-item questionnaire, women reported the severity of the event's impact during the prior 12 months (possible item score range 1 “not at all” to 5 “extremely”). The reported impact of all endorsed events was summed to generate a weighted score (range: 0–100 in our sample).^32^

#### Stress in pregnancy

Stressful life events during the index pregnancy were assessed with the Crisis in Family Systems-Revised (CRISYS-R) survey,^33^ which is validated in English and Spanish and has demonstrated validity and reliability in socioeconomically diverse and pregnant populations.^34,35^ The survey assesses 11 domains: financial, legal, career, relationships, medical issues (self), medical issues (others), community safety, home safety, housing problems, difficulty with authority, and discrimination. Respondents report whether each listed event occurred in the prior 6 months and, for each endorsed event, whether it was positive, negative, or neutral. An NLE domain score was generated by summing the number of domains in which negative events were reported (range: 0–9 in our sample).^29^

#### Psychological symptoms in pregnancy

Depressive symptoms were assessed with the Edinburgh Postnatal Depression Scale (EPDS),^36^ validated against clinical evaluation in prenatal and postnatal contexts.^37,38^ The scale has 10 items referring to experiences over the previous 7 days, each scored 0–3. Two items are reverse-coded, and then, the 10 responses summed to create a unidimensional depressive symptom score, with higher values indicating greater symptomatology (range: 0–26 in this sample).

PTSD symptoms were assessed with the 17-item PTSD Checklist-Civilian version (PCL-C),^39^ which has demonstrated reliability and validity with clinical diagnosis.^40^ Participants rated the extent to which they were bothered by each listed symptom in the prior month, with possible responses ranging from 1 (not at all) to 5 (extremely). Responses were summed to create a score, with higher values indicating greater reported PTSD symptoms (range: 16–85 in this sample).

### Outcome

Infant temperament at age 6 months was assessed with the Infant Behavior Questionnaire—Revised (IBQ-R) designed to measure temperament between the ages of 3 and 12 months.^24,41^ Mothers rated the frequency with which their infant demonstrated listed behaviors over the prior week, rating each of 191 items on a 7-point scale from 1 (never) to 7 (always). Scores were summed according to the IBQ-R guidelines to create 14 behavioral domain scales. Prior research has identified 3 overarching groupings of the 14 domain scales,^24^ which we confirmed in our sample.^42^ “Negative affectivity” comprises four scales: Distress to Limitations/Frustration, Falling Reactivity/Rate of Recovery, Fear, and Sadness. NA was calculated by taking the mean value of the four component scores, with Falling Reactivity reverse-coded.^24^ NA ranged from 1.4 to 5.4 in this sample, with higher values indicating more frequent and intense expressions of NA.

### Covariates

Maternal sociodemographics were measured with questionnaires at enrollment. Women self-reported their race/ethnicity, which was then collapsed into four categories: white, black, Hispanic, and other. Women who identified as black were categorized in that group regardless of whether they also identified as Hispanic. Maternal education was queried as the highest level of school completed, with five possible answers ranging from less than 12th grade to graduate degree. For this analysis, mother's education was dichotomized into low (less than a high school degree) and high (high school degree or higher). The infant's sex and date of birth were reported by the mother in the first postnatal interview.

### Statistical analysis

Maternal/infant dyads with complete prenatal maternal stress data and infant temperament assessments at age 6 months were included in the analyses (*n*=445). Those included did not differ on sociodemographic characteristics (mother's age, education, race/ethnicity, infant sex) from those missing exposure or outcome data.

Correlations among the maternal stress variables were investigated with Pearson correlations. To determine the association between the four maternal stress components—continuous traumatic and nontraumatic lifetime stressors weighted score (LSC-R), NLEs, depressive symptoms (EPDS), and PTSD symptoms—and infant NA, analyses proceeded in two phases. First, separate multivariable linear regression models were developed to evaluate the association between each maternal stress component and NA. Models were adjusted for mother's race/ethnicity, education level, and infant sex. Nonlinear associations were investigated with quadratic terms for the stress variables; evidence of nonlinearity was not observed, and so, the quadratic terms were ultimately not retained in final models. *p* Values were adjusted for multiple comparisons with a Bonferroni correction.

Next, WQS regression analysis, a supervised machine-learning technique, was used to investigate the relationship between the prenatal stress “mixture” and NA. The WQS calculates a mixture index comprising the weighted sum scores of quantiled (here, quartiles) mixture components—LSC-R, NLE, EPDS, and PTSD scores—to test the association between the mixture of stressors and the outcome variable, NA. Higher scores on the WQS index reflect greater stress. WQS regression evaluates the effect of the mixture index in a traditional linear regression model, providing a coefficient, standard error, and *p*-value for the association between the mixture index and NA adjusted for specified covariates. The method additionally provides weights for each stress component, which convey the extent to which each contributes to the relationship between the mixture and the outcome. To evaluate the extent to which the stress components driving the association with infant NA were dependent on maternal race/ethnicity, product terms were introduced into the WQS model for each stress measure. This creates race-specific weights, such that we can evaluate the differential effect of each stressor in each racial/ethnic group. Due to sample size constraints, the “other” race group was omitted from the analysis.

For more details of the WQS regression method, see Ref.^23^ Briefly, the WQS model takes the form as follows:

(1) $$g\left(\mu \right)=\beta_{0}+\beta_{1}\left(\sum_{i=1}^{c}w_{i}q_{i,j}\right)+z\prime \varphi ,$$

where *g*(*μ*) is the identity link function for continuous outcome infant NA scale score, *q_i_* is the quantile score for mixture component *I*, participant $j,w_{i}\left(0<w_{i}<1,\sum_{i=1}^{c}w_{i}=1\right)$ is the weight parameter for the *i*th component, and *z* is a vector of covariates. Then, the term $\sum_{i=1}^{c}\bar{w_{i}}q_{i}$ is the weighted score for the set of mixture components c, with mean estimated weights *w_i_* determined through maximum likelihood estimation in a specified number of bootstrapped samples. The data set was split for testing and validation to avoid overfitting, with 60% of observations withheld for validation. *N*=250 bootstrap samples were used to estimate weights. All analyses were conducted in R,^43^ version 3.5.3, using the *qWQS* package.

## Results

### Sample characteristics

Table 1 displays participant sociodemographic characteristics. As shown in Table 1, the majority of women self-identified as black or Hispanic. Stress and psychological symptom scores in pregnancy were similar in black and Hispanic women, and scores in minority subgroups were somewhat higher than in white women (analysis of variance [ANOVA] with Bonferroni-adjusted *p*<0.05 for all stress measures comparing black with white women and Hispanic with white women). A similar pattern was observed for infant NA; although small in magnitude, NA scores were significantly different when comparing infants of black versus white and Hispanic versus white women (ANOVA Bonferroni-adjusted *p* for all <0.01). Correlations among stress measures confirmed moderately strong positive associations among stress variables (Table 2). This collinearity precluded developing a multivariable linear regression with the four stress predictors in the same model. Correlation coefficients were largely unchanged when recalculated stratified by maternal race/ethnicity (not shown).

**Table 1.** Participant Sociodemographic, Stress Exposure, and Temperament Characteristics (*n*=445)

Characteristic	Overall	Black	Hispanic	White
	% (n)			
Maternal race/ethnicity				
Black	44 (196)			
Hispanic	37 (163)			
White	19 (86)			
Mother's education				
High school or greater	59 (263)	56 (109)	44 (71)	97 (83)
Less than high school	41 (182)	44 (87)	56 (92)	3 (3)
Infant sex				
Female	46 (206)	46 (91)	48 (79)	42 (36)
Male	54 (239)	54 (105)	52 (84)	58 (50)
	Median (IQR)			
Lifetime stress/trauma (LSC-R)	10 (12)	12.5 (15.2)	11 (12)	6.5 (5.8)
NLEs in pregnancy (CRISYS-R)	2 (3)	3 (3)	2 (3)	1 (2.8)
Depressive symptoms in pregnancy (EPDS)	5 (8)	6 (9)	5 (9)	4 (7)
PTSD symptoms in pregnancy (PCL-C)	19 (10)	20 (13)	19 (10)	17 (5)
Infant NA scale score (IBQ-R)	3.1 (0.9)	3.1 (0.9)	3.2 (0.9)	2.8 (0.7)

CRISYS-R, Crisis in Family Systems—Revised; EPDS, Edinburgh Postnatal Depression Scale; IBQ-R, Infant Behavior Questionnaire—Revised; LSC-R, Life Stressor Checklist—Revised; NA, negative affectivity; NLE, negative life event; PTSD, post-traumatic stress disorder; PCL-C, PTSD Checklist—Civilian version.

**Table 2.** Correlations Among Maternal Stress and Psychological Symptom Measures in Pregnancy

Stress domain	LSC-R	NLE	EPDS
NLE	0.51		
EPDS	0.37	0.37	
PTSD	0.51	0.31	0.39

Values are Pearson correlation coefficients. All correlations are significant at *p*<0.01.

NLE, negative life events (assessed with Crisis in Family Systems, Revised—CRISYS-R); PTSD, post-traumatic stress disorder symptoms assessed with PCL-C.

### Linear regression models

**FIG. 1.**
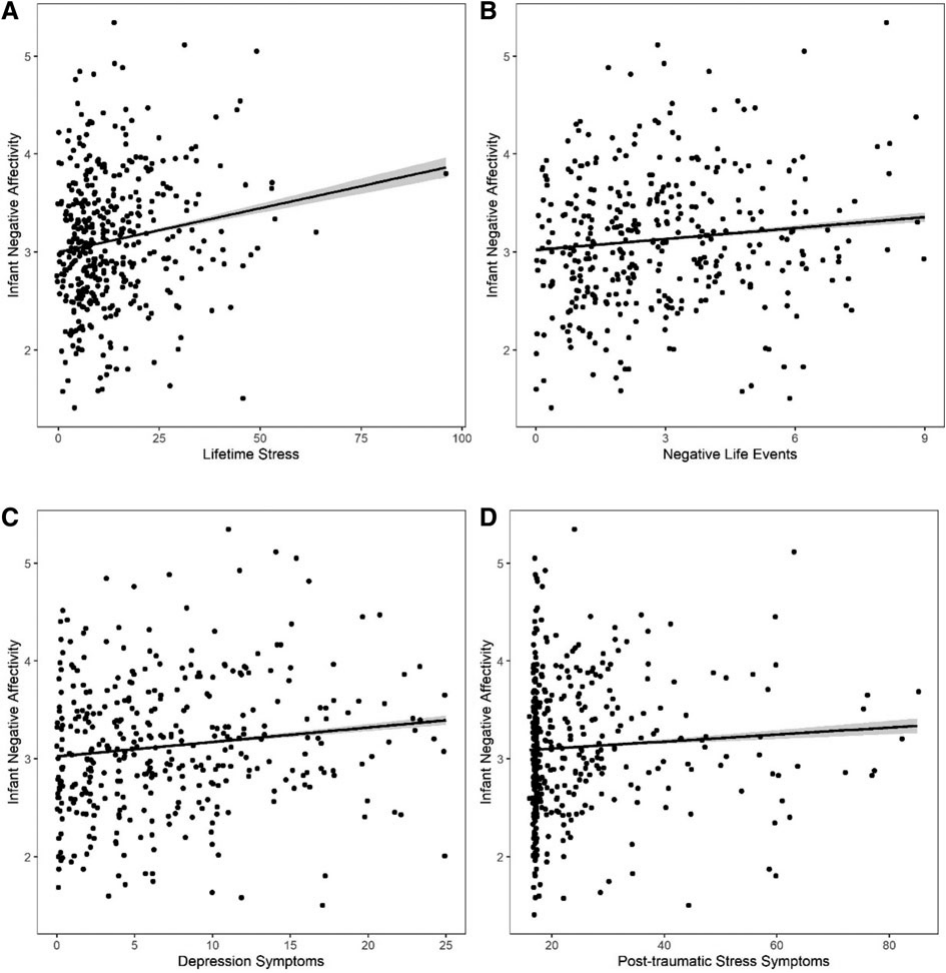
Associations between infant NA scale score and each maternal stress and psychological dysfunction measure according to adjusted linear regression models: lifetime stress [predicted value of the dependent variable in the equation (*y*)=0.007*x*+2.963; *p*-value=0.008] **(A)**, prenatal negative life events (*y*=0.031*x*+2.990; *p*-value=0.043) **(B)**, prenatal depression symptoms (*y*=0.0136*x*+2.989; *p-*value=0.010) **(C)**, and prenatal PTSD symptoms (*y*=0.004*x*+2.985; *p*-value=0.140) **(D)**. Models are adjusted for mother's education level, race/ethnicity, and infant sex. *Shaded areas* denote 95% CI. CI, confidence interval; NA, negative affectivity; PTSD, post-traumatic stress disorder.

Associations between individual stressors and NA assessed in discrete linear models are shown in Figure 1. We found that maternal lifetime stress, NLEs, and symptoms of depression and PTSD, each considered in separate models, were positively associated with infant NA. Coefficients for the individual stressors were statistically significant for all but PTSD in models adjusted for maternal race/ethnicity and education and infant sex.

### WQS analysis

**FIG. 2.**
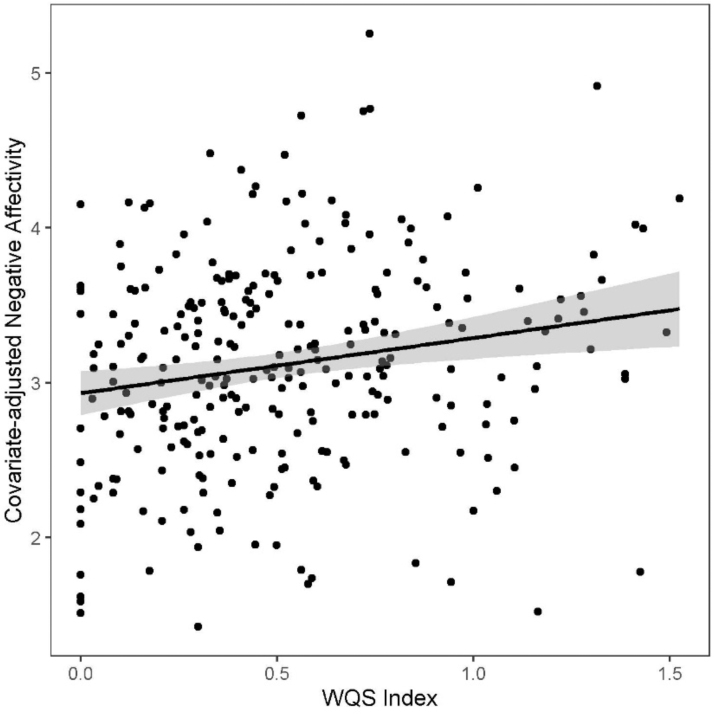
Scatterplot and linear fit line with 95% CI for association between covariate-adjusted infant NA and WQS stress score for mother/infant dyads. Model is adjusted for maternal education, race/ethnicity, and infant sex. WQS, weighted quantile sum.

**FIG. 3.**
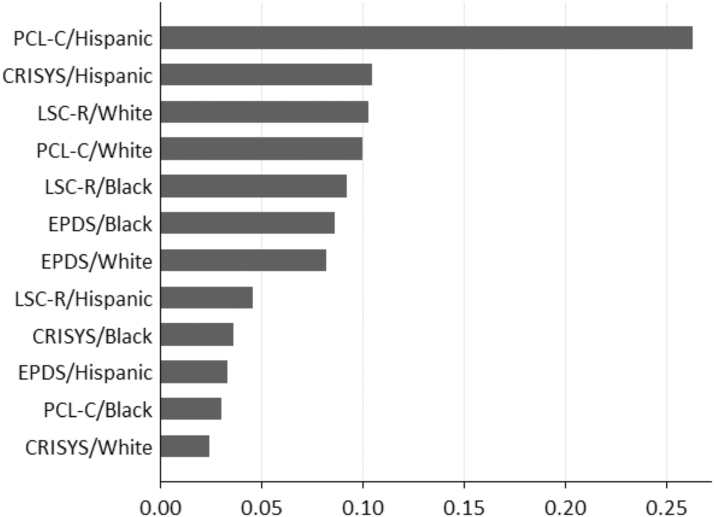
Race/ethnicity-specific mixture weights for the WQS regression index of maternal stress in pregnancy on infant NA. CRISYS, Crisis in Family Systems, Revised (measures negative life events); EPDS, Edinburgh Postnatal Depression Scale; LSC-R, Life Stressor Checklist—Revised; PCL-C, PTSD Checklist—Civilian version.

We next tested the combined effects of multiple stressors in a WQS mixtures model with race-specific weighting. This approach allowed us to evaluate the relative contribution of stressors in each racial/ethnic group to the overall mixture effect. In the sample as a whole, there was a significant positive linear association between the prenatal weighted stress index and infant NA (Fig. 2). For each one-unit increase in the weighted prenatal stress index, the infant NA score increased by 0.40 (0.16–0.64) (*β* [95% confidence interval]). When quantifying the contribution of each stress component in the mixture conditional on race/ethnicity (i.e., weights sum to 100% within the race/ethnic group), the weight distributions varied across groups (Table 3, Fig. 3). Among Hispanic women, the association between greater maternal prenatal stress and higher NA in infants was driven by greater PTSD symptoms (59%) and NLEs in pregnancy (24%). In black women, lifetime traumatic and nontraumatic events (38%) and greater depressive symptoms (35%) accounted for the majority of the weight. In contrast to black and Hispanic women, conditional weights in white women were almost equally distributed across lifetime traumatic and nontraumatic stressors (33%), depressive symptoms (27%), and PTSD symptoms (32%).

**Table 3.** Race/Ethnicity-Specific Mixture Weights for the Weighted Quantile Sum Regression Index of Maternal Stress in Pregnancy on Infant Negative Affectivity

Race/stress domain	Weight	Conditional weight^a^
Black		
Lifetime stress/trauma (LSC-R)	0.092	0.376
NLEs in pregnancy (CRISYS-R)	0.036	0.149
Depressive symptoms in pregnancy (EPDS)	0.086	0.351
PTSD symptoms in pregnancy (PCL-C)	0.030	0.124
Hispanic		
Lifetime stress/trauma (LSC-R)	0.046	0.103
NLEs in pregnancy (CRISYS-R)	0.105	0.235
Depressive symptoms in pregnancy (EPDS)	0.033	0.075
PTSD symptoms in pregnancy (PCL-C)	0.263	0.588
White		
Lifetime stress/trauma (LSC-R)	0.103	0.333
NLEs in pregnancy (CRISYS-R)	0.024	0.078
Depressive symptoms in pregnancy (EPDS)	0.082	0.266
PTSD symptoms in pregnancy (PCL-C)	0.100	0.323

^a^ Conditional weights are relative weights within each race/ethnicity stratum.

PTSD, PTSD symptoms assessed with the PCL-C.

## Discussion

Individual measures of maternal stress and psychological dysfunction in pregnancy, including depression and anxiety,^30,44^ PTSD,^45,46^ and interpersonal violence,^47^ have been linked to infant and childhood temperament in prior studies. We extend methods traditionally used to study the health effects of environmental chemical mixtures to quantify the total association between a set of correlated stress and psychological symptom measures in pregnant women in relation to their infant's propensity to NA. Higher scores on the integrated index of prenatal stress were associated with greater infant NA. Furthermore, in this diverse urban sample, we found that the individual contribution of each stress component of the mixture was different when conditioned on maternal race/ethnicity. These findings have implications for improved targeting of maternal mental health screening and service delivery in the context of prenatal health care. They can additionally inform future studies in that important determinants of infant NA and perhaps other elements of development could be overlooked if differences in salient risk factors by maternal race and ethnicity are not considered.

These analyses add to an evolving literature documenting racial/ethnic differences in the experience of stress and the psychological embedding of stress effects. For example, Brown et al. reported greater distress among whites than blacks and Hispanics following the same degree of self-reported stress exposures in specific domains.^48^ In contrast, Liu et al. found greater risk of depressive symptoms among black and Hispanic women who experienced interpersonal and financial stress during pregnancy, compared with white women.^49^ These findings also build on a prior study in the same cohort that used WQS regression to examine associations between a prenatal stress “mixture” and a biomarker of placental oxidative stress, mitochondrial DNA copy number (mtDNAcn).^50^ Brunst et al. found that higher scores on the prenatal stress mixture index were associated with reduced mtDNAcn, reflecting increased placental oxidative stress, and that white and no-white women differed in the manifestation of psychological dysfunction that most accounted for the relationship with infant NA. The current analyses extend those findings to a functional outcome in infants. Furthermore, our larger analytic sample allowed us to examine black and Hispanic women separately, corroborating prior studies suggesting that relevant stress exposures and consequent psychological responding may differ among US black, Hispanic, and white women.^49,51–53^

Notably, research suggests that NA may be relatively stable across the life course and has implications for later adverse outcomes. For example, NA domains (fear, sadness, distress reactivity, and recovery) assessed in infants predict similar measures of NA assessed in later childhood, as well as adult personality domains such as neuroticism.^24^ Elevated NA has also been linked with increased externalizing behaviors,^29^ sleep disorders,^54^ and depressive symptoms later in life.^55^ Elucidating factors that contribute to NA in infancy may inform future interventions that can promote a more optimal developmental trajectory. Our findings suggest that interventions aimed to mitigate effects of prenatal maternal stress on offspring developmental outcomes will be more effective if tailored to account for observed racial/ethnic differences, which has been noted in other contexts.^56,57^

While clinicians may screen for current ongoing adversities in pregnant women under their care, they do not typically screen for lifetime cumulative exposures, including traumatic experiences. It is only recently that health care providers have recognized the prevalence of traumatic stressors across the life course and the impact they have on a range of health outcomes. The need to screen for lifetime traumas and deliver trauma-informed care is increasingly recognized.^58^ Moreover, pregnancy is a critical time to identify and address maternal mental health problems, for the health of both mother and child. Historically, the most commonly recognized perinatal mental health disorders have been depression and anxiety, with an estimated prevalence ranging from 6.5% to 14% for depression or depressive symptoms,^59^ and 13% to 16% for anxiety disorders or symptoms.^60^ More recently, epidemiologic surveillance data highlight PTSD as a significant mental health concern during pregnancy.^61^ While routine screening for perinatal depression and anxiety disorders in obstetrics and gynecology settings is becoming more widespread,^62^ screening for PTSD is not routine. These findings underscore the need for clinicians to gain insight into the cumulative stress experiences in women under their care as well as screen broadly for psychological dysfunction.

We note a number of strengths as well as acknowledge some weaknesses of the current analyses. Strengths of the present study include the large racially/ethnically diverse sample of mother/infant dyads enrolled prenatally and the assessment of stress and psychological dysfunction across multiple domains during pregnancy using validated questionnaires. We were able to leverage this data set to extend methods traditionally used to study the health effects of environmental chemical mixtures that co-occur and may be highly correlated,^21,22^ to more comprehensively examine the individual and joint relationships between multiple correlated maternal lifetime and prenatal stress-related measures and infant NA. The WQS method groups each exposure variable into quartiles, which could result in some loss of information relative to analyzing continuous data, protects against potential misestimating of effects due to outliers or extreme values, and provides an across-scale normalization. The WQS method, like a typical linear model, is associative and in the context of an observational study cannot be considered informative from a causal perspective. Additional research is needed to corroborate these findings and extend them to other cohorts with different racial/ethnic and socioeconomic makeups.

The IBQ-R, used to measure infant NA, is a widely used and validated measure, including demonstrated correlations with laboratory measures.^41,63^ Also, we previously confirmed the factor structure in our diverse sample.^42^ Parent-report measures of infant temperament have recognized benefits, including providing a low-cost method for obtaining information on a large sample, and they leverage the mother's ability to observe her infant's behavior over numerous contexts that are not affected by the artificiality of structured laboratory observations.^64^ However, reliance on maternal report increases risk for diminished validity due to the potential impact of maternal psychopathology on the accurate perception and reporting of child behaviors. This concern is diminished in this prospective study in which maternal psychological symptoms are assessed in pregnancy and the child outcome is assessed ∼6 months postnatally. Moreover, the IBQ-R was designed to reduce the influence of reporter biases by inquiring about concrete infant behaviors rather than abstract judgments.^24^ Nonetheless, future studies could consider the use of more objective measures of infant behavior, including naturalistic home and structured laboratory observations.

This study did not consider postnatal maternal psychological functioning, which has been associated with infant NA. Notably, data suggest that prenatal exposures exert effects on infant emotional functioning independent of postnatal factors.^30,65,66^ In addition, using a life course framework, we anticipate that there will be cumulative effects of pre/postnatal psychopathology, which may start to program infant neurodevelopment through physiological disruption (e.g., differential cortisol production), whereas postnatal maternal psychological functioning may operate through other pathways such as infant/caregiver interactions. The examination of these cumulative effects is beyond the scope of this article.

## Conclusion

This study demonstrates the application of a supervised machine-learning technique to relate experiences of maternal lifetime and prenatal NLEs and psychological dysfunction in pregnancy to infant NA, and offers new insights into the etiology of differences in prenatal stress effects by maternal race and ethnicity. The relative contribution of stress elements differed across racial/ethnic groups, which highlights that studies examining a single stress measure may miss or obscure underlying associations between maternal stress and infant neurobehavioral development in diverse populations. Implementing tailored statistical methods that allow for the study of multiple correlated stress measures together with psychological sequelae, accounting for effect modification by race/ethnicity, may better elucidate those at increased risk for intergenerational stress effects on adverse neurobehavioral outcomes in early childhood and better guide effective intervention approaches. These findings also have implications for broader screening for lifetime experiences of trauma as well as ongoing NLEs in pregnant women, as well as broader screening for mental health symptoms beyond the current focus on anxiety and depressive symptoms. Trauma-informed prenatal health care and screening, including PTSD symptomatology, need to become routine. Intervening around the mother's experiencing of cumulative stress/trauma as well as her psychological functioning may be the best way to put the child on an optimal trajectory.

## Abbreviations Used

ANOVA: analysis of variance
CI: confidence interval
EPDS: Edinburgh Postnatal Depression Scale
IBQ-R: Infant Behavior Questionnaire—Revised
LSC-R: Life Stressor Checklist—Revised
mtDNAcn: mitochondrial DNA copy number
NA: negative affectivity
NLE: negative life event
PTSD: post-traumatic stress disorder
WQS: weighted quantile sum
